# The effects of acupuncture on clinical efficacy and steady-state visual evoked potentials in insomnia patients with emotional disorders: A randomized single-blind sham-controlled trial

**DOI:** 10.3389/fneur.2022.1053642

**Published:** 2023-01-18

**Authors:** Leixiao Zhang, Yanli Deng, Ruting Hui, Yu Tang, Siyi Yu, Ying Li, Youping Hu, Ning Li

**Affiliations:** ^1^Department of Integrated Traditional and Western Medicine, West China Hospital, Sichuan University, Chengdu, China; ^2^Sichuan Second Chinese Medicine Hospital, Chengdu, China; ^3^Chengdu First People's Hospital, Chengdu, China; ^4^Chongqing Emergency Medical Center, Chongqing, China; ^5^Acupuncture and Tuina School, Chengdu University of Traditional Chinese Medicine, Chengdu, China

**Keywords:** acupuncture, sham acupuncture, insomnia, anxiety, depression, SSVEP, electroencephalogram

## Abstract

The aim of this study was to observe the clinical effects and brain electrical potential changes following acupuncture in the treatment of insomnia patients with mood disorders. Ninety patients with insomnia who met the inclusion criteria were randomly divided into the active acupuncture group (AA group, *n* = 44) and sham acupuncture group (SA group, *n* = 46) at a ratio of 1:1. The primary outcome was the total score of the Pittsburgh Sleep Quality Index (PSQI), and the secondary outcomes were the total effective rate, Self-Rating Anxiety Scale (SAS), Self-Rating Depression Scale (SDS) scores, and values of steady-state visual evoked potentials (SSVEP). The two groups received acupuncture or sham acupuncture 10 times (2 weeks). Finally, the total PSQI scores of the AA group and SA group were significantly different (*p* < 0.05) at 2 weeks (6.11 ± 2.33 vs. 10.37 ± 4.73), 6 weeks (6.27 ± 1.39 vs. 11.93 ± 3.07), 18 weeks (6.32 ± 2.84 vs. 11.78 ± 2.95) and 42 weeks (8.05 ± 3.14 vs. 12.54 ± 2.81). Further analysis found that AA group patients received acupuncture treatment at any age after the same effect (*p* > 0.05). The SAS and SDS scores of the AA group were also significantly different from those of the SA group at each assessment time point (*p* < 0.05). The total effective rate of the AA group was 81.82%, while that of the SA group was 30.43% (*p* < 0.05). There was no significant difference between the AA group and SA group only in the brain potential of the parietal lobe (F4), left temporal lobe (C3) and right temporal lobe (T8) (*P* > 0.05), but there was a significant difference between other brain regions (*P* < 0.05). In addition, correlation analysis showed that there was a certain positive correlation between the total PSQI score, SAS score, efficacy level, and SSVEP value in the AA group as follows: C4 and the total PSQI score (*r* = 0.595, *P* = 0.041), F3 and SAS score (*r* = 0.604, *P* = 0.037), FPz and efficiency level of the frontal lobe (*r* = 0.581, *P* = 0.048), and O2 and efficiency level of the occipital lobe (*r* = 0.704, *P* = 0.011). Therefore, acupuncture have a good clinical effect on patients with insomnia and emotional disorders and have a significant regulatory effect on abnormally excited brain potentials.

## 1. Introduction

Insomnia is a painful and disabling condition that affects a large proportion of the general population. It is a risk factor for impaired functioning and other medical and mental disorders (1). Approximately 10–20% of the world's population suffers from insomnia, and there has been an increased incidence of insomnia worldwide (2). The prevalence of insomnia among Chinese adults reaches up to 19.6% (2). Moreover, insomnia is 1.5 times more common in women than in men (3). Long-term chronic insomnia can cause major health problems and other chronic diseases, such as diabetes (4), obesity (5), and mood disorders (6). Insomnia may be a symptom of other diseases (e.g., stroke) and often coexists with mental and physical health conditions (7).

Insomnia is a cross-diagnostic symptom associated with the development of mental disorders and is most closely related to depression (8). While developing sleep disorders, depression also has a positive influence on mood adjustment (9). The economic and treatment costs for insomnia are also high, totaling more than 100 billion US dollars each year. The indirect costs of productivity losses caused by insomnia include lower work performance, increased healthcare utilization, and increased accident risks (10). Psychological treatment methods such as cognitive behavioral therapy for insomnia (CBT-I) are internationally recommended treatment options for insomnia.

Further evidence from meta-analysis results has shown that compared with fake acupuncture/placebo acupuncture or the effects of waiting for treatment, acupuncture can increase total sleep time, improve sleep efficiency, reduce wakefulness after falling asleep, and reduce the number of awakenings. (11). In addition, the results from our previous research have also confirmed that acupuncture can significantly improve the sleep quality of patients with insomnia affected by mood disorders and that the posttreatment effect can last for more than 6 weeks (12).

However, these issues remain unexplored in the literature. Previous studies have shown that the efficacy evaluation of acupuncture for insomnia is still mainly based on the scale, but there have been few studies using steady-state visual evoked potential (SSVEP) as an objective indicator to evaluate the improvement of sleep quality. Brain computer interface (BCI) technology provides a new cognitive channel for human beings by encoding and decoding brain activity. SSVEP-based BCI stands out from other BCI paradigms because of its advantages of being noninvasive, requiring little user training, and yielding a high information transfer rate (13). SSVEP are widely used in electroencephalogram (EEG) responses elicited by periodic visual stimuli. It has also been widely used to record mood changes (14) as a research tool in social and emotional neuroscience (15).

Much remains to be known about the key mechanisms regulating the cerebral cortex. Therefore, to determine whether acupuncture is an effective treatment option for patients with insomnia, a technique is needed to track the transient electrophysiological effects of acupuncture and to record the cortex's response to electrophysiological changes. Based on the above discussion, we observed the clinical efficacy and brain electrical potential changes of patients with insomnia and emotional disorders treated with acupuncture for the first time, taking the brain potential based on the SSVEP as an indicator and combining it with the clinical scale.

## 2. Research methods

### 2.1. Ethics and design

The design and reporting of our controlled trials conformed to the following principles: The Uniform Standards for Reporting Trials (CONSORT) and the Standards for Reporting Interventions in Acupuncture Clinical Trials (STRICTA) Guidelines (16, 17). After obtaining ethical approval from the Sichuan Traditional Chinese Medicine (TCM) Regional Ethical Review Committee (No. 2013KL-016), we started to recruit patients for the study. Due to the maintenance of the Chinese clinical trial registration website, this study was reregistered on October 18, 2018 (ChiCTR1800018958). All patients or authorized guardians signed written informed consent forms.

### 2.2. Participants

The insomnia patients in this study were recruited through outpatient advertisements and recommendations. All patients received free treatment in the Acupuncture Department of Sichuan Provincial Hospital of Traditional Chinese Medicine in China between May of 2014 and August of 2016. The diagnostic criteria for patients with insomnia are based on the “Diagnostic and Statistical Manual 5 criteria” (18).

Inclusion criteria were as follows: patients who met the diagnostic criteria for insomnia (male or female 18–65 years old); patients in whom insomnia symptoms occurred at least 3 times a week and lasted for more than 1 month; and patients with insomnia who had not taken any anti-insomnia drugs before. There were notable symptoms of anxiety and depression or withdrawal anxiety in patients for more than 7 days (PSQI score >7, SAS score ≥50, SDS score ≥50). Informed consent was obtained from the patients.

Exclusion criteria were as follows: patients who had systemic diseases such as psychosomatic disorder, fever, cough or serious diseases involving vital organs and the haematopoietic system; patients who were alcohol- and drug-dependent or were pregnant or lactating; and patients who participated in other studies.

### 2.3. Sample size calculation

Previous studies (19) have found a difference of at least 2.7 points in PSQI scores between active acupuncture treatment and sham acupuncture treatment. We expected a 3.2 point difference in active vs. sham acupuncture treatment in this study. Therefore, at the significance level of 0.05, the difference in detection ability between the two groups was 95%, and the sample size was 40 in each group. Considering a drop-out rate of 10%, the total sample size required was 44 for each group in this study.

### 2.4. Randomization and blinding

The random number table was generated using SPSS 22.0^®^ software. The random Arabic numeral 0 represents the active acupuncture (AA) group, and 1 represents the sham acupuncture (SA) group. The random distribution cards containing random numbers, serial numbers, and groups and were put in opaque, serial-numbered, leather envelopes. Insomnia patients then unsealed the envelopes after meeting the inclusion criteria according to the order of visit. The included patients were randomly divided into the AA and SA groups at a ratio of 1:1. This random assignment is single-blind, and the patients do not know which group they are in. Due to the particularity of the acupuncture therapy, acupuncture treatment allocation was concealed from everyone (e.g., evaluators and data processing statisticians) except the acupuncturist.

### 2.5. Intervention protocols

**Figure 1:**
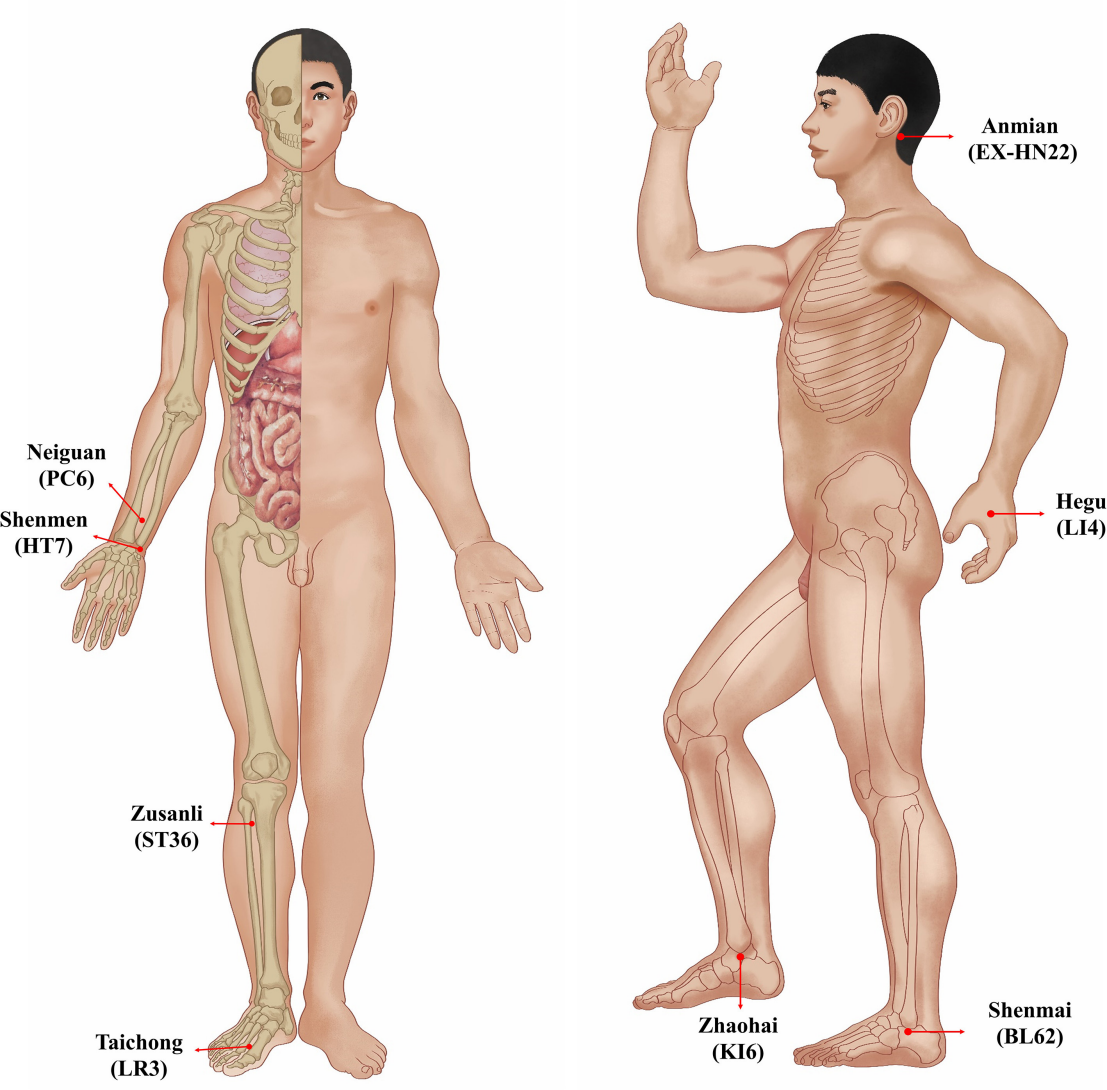
Location of acupoints.

**Figure 2:**
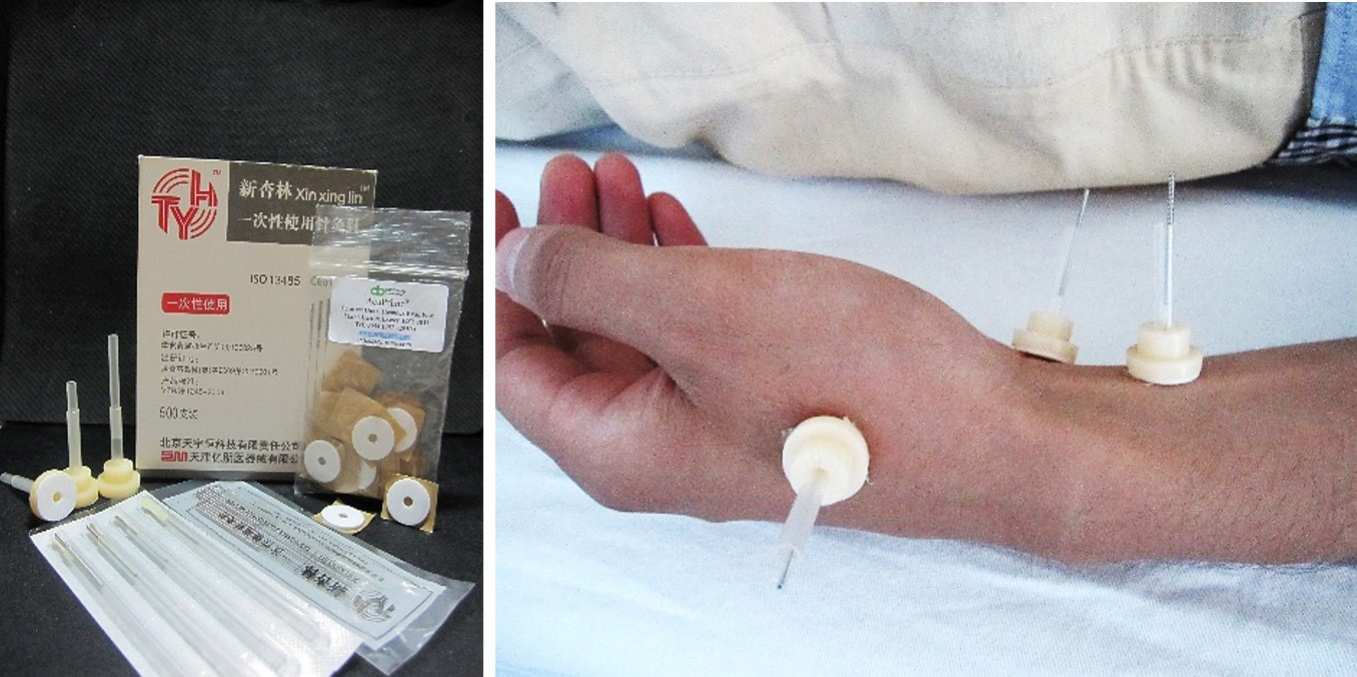
The active or sham acupuncture intervention (PSD, real an d blunt needles).

The acupuncture treatments in this study were all completed by the same acupuncturist, who had a master's degree and at least 5 years of clinical experience. The acupuncturist chose the same acupuncture points in the patients from both the AA group and the SA group (Table 1, Figure 1). These acupuncture points included bilateral Anmian (EX-HN22), Neiguan (PC6), Shenmen (HT7), Hegu (LI4), Zusanli (ST36), Zhaohai (KI6), Shenmai (BL62) and Taichong (LR3). The acupuncturist used the Park Sham Acupuncture Device (the PSD) to match real needles and blunt needles in both groups (the PSD approval number: 9018390000, DONGBANG Acupuncture Inc.) (Figure 2). The 8 acupoints we selected were artificially stimulated every 15 min, and the duration of each acupoint was 5 s. Patients are treated once a day for 30 min. The treatment was continued for five consecutive days, followed by 2 days of rest. Both the AA group and the SA group received 10 acupuncture (2 weeks) treatments. The follow-up period was half a year, with assessments completed at 6, 18, and 42 weeks.

**Table 1:** Description of each acupoint and acupuncture method.

**No**.	**Acupoint**	**Description of acupoints**	**Needling method**	**Needles**
1	Anmian (EX-HN22)	The midpoint between the depression behind the ear and the suboccipital depression.	The needle is punctured 0.5–0.8 cun vertically	0.25^*^25 mm
2	Neiguan (PC6)	Between the tendons of palmaris longus and flexor carpi radialis, 2 cun above the transverse crease of the wrist.	The needle is punctured 0.5–0.8 cun vertically	0.25^*^25 mm
3	Shenmen (HT7)	On the palmar ulnar end of the transverse crease of the wrist, and on the radial aspect of the tendon of the ulnar flexor of the wrist.	The needle is punctured 0.3–0.5 cun vertically	0.25^*^25 mm
4	Hegu (LI4)	Between the 1st and 2nd metacarpal bones, and in the midpoint of the radial side of the 2nd metacarpal bone.	The needle is punctured 0.5–0.8 cun vertically	0.25^*^25 mm
5	Zusanli (ST36)	3 cun directly below lateral depression of the patella ligament, and one finger-breadth lateral to the anterior border of the tibia.	The needle is punctured 1.0–1.3 cun vertically	0.25^*^40 mm
6	Zhaohai (KI6)	In the depression directly below the tip of the medial malleolus.	The needle is punctured 0.2–0.5 cun vertically	0.25^*^25 mm
7	Shenmai (BL62)	In the depression directly below the tip of the lateral malleolus.	The needle is punctured 0.2–0.5 cun vertically	0.25^*^25 mm
8	Taichong (LR3)	In the depression anterior to the junction of 1st and 2nd metatarsal bones.	The needle is punctured 0.3–0.5 cun vertically	0.25^*^25 mm

**Figure 3:**
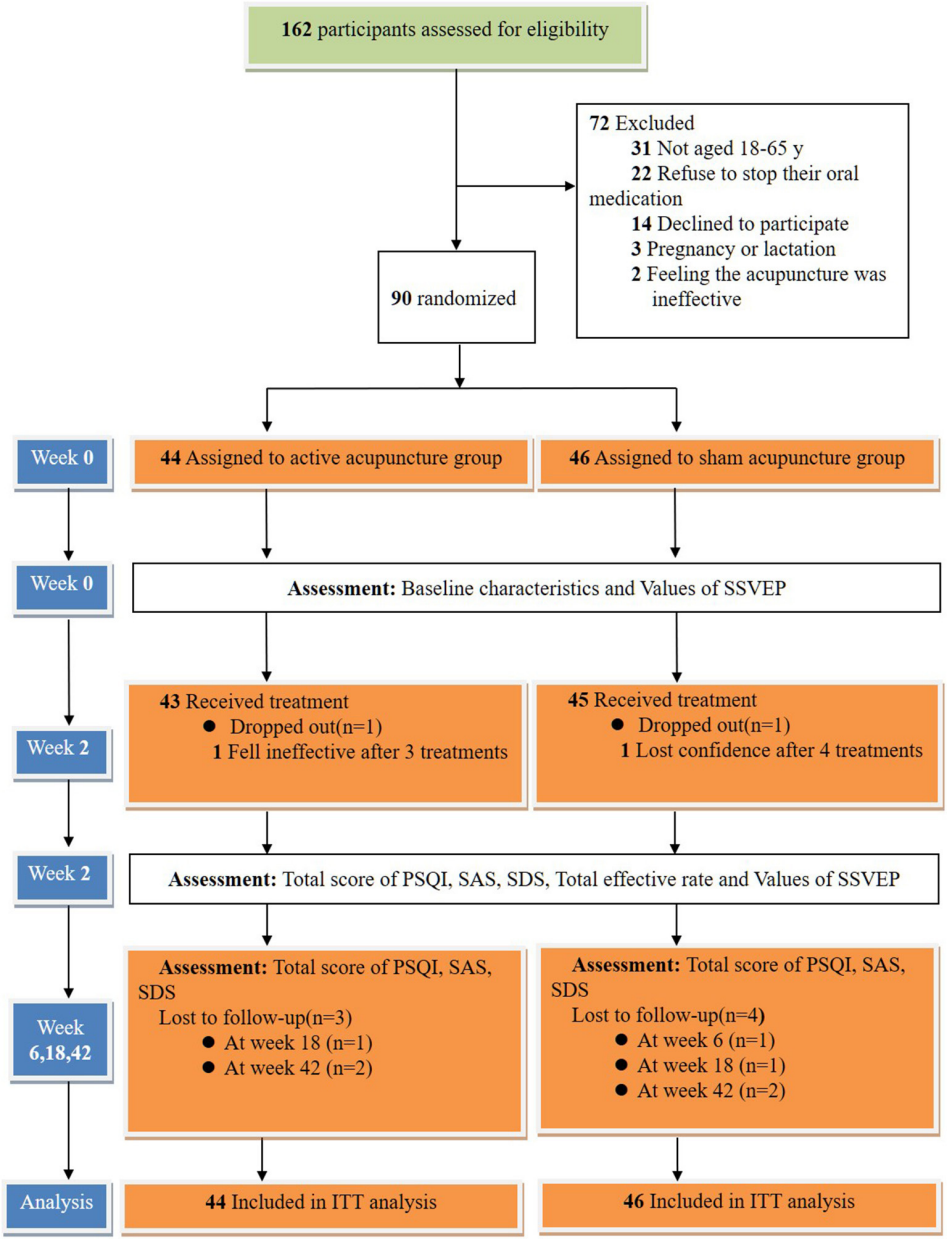
Trial flow diagram (The Screening, Enrolment, Randomization, Interventions, Follow-up, and Analysis). ITT indicates intention to treat.

Patients were not allowed to take any sleep medications during the entire study period. However, patients who could not tolerate sleep disorders in special circumstances were allowed to take estazolam tablets as a rescue medication (0.5–2 mg, approval number: H37023047, Shandong Xinyi Pharmaceutical Co., Ltd.) before going to bed. After their condition was relieved, the patients were asked to stop taking the medication immediately. Their medications were recorded in a notebook. The trial flow diagram is shown in Figure 3.

#### 2.5.1. Active acupuncture group

We allowed patients to lie quietly in bed during acupuncture treatment. After the acupuncturist disinfected their hands and the patients' acupoint skin with 75% alcohol, the Xin Xinglin disposable needle (0.30 × 25 mm/0.30 × 40 mm) was used, together with the PSD (12). The acupuncturist exposed the needle tip and used a suitable insertion method to penetrate the needle through the skin at acupuncture points while sticking the PSD to the skin at the same time. The angle and depth of needle insertion into each acupoint are shown in Table 1. During the entire 30 min needle retention period, the acupuncturist rotated or lifted the needle every 10 min to achieve the “deqi” sensation (pain, heaviness, numbness, swelling or radiation).

#### 2.5.2. Sham acupuncture group

The acupuncturist used the PSD combined with a blunt needle (0.30 × 25 mm/0.30 × 40 mm) in treating the patients in the SA group. Blunt needle is composed of a hollow needle shaft that can be stretched and a blunt needle head (20). The acupuncturist completed the same disinfection process as used in the AA group and then tapped the blunt needle of the PSD gently against the surface of the skin at the acupuncture points. The blunt needle was not manipulated to minimize any physiological effects. In the SA group, unless the patients did not experience “deqi”, the operations were the same as those conducted in the AA group, including acupoint selection, needle placement times, and needle withdrawal methods (21).

### 2.6. Primary outcomes

We completed PSQI assessments at baseline, after treatment (week 2), and during follow-up (weeks 6, 18, and 42). Each observation index is scored from 0 to 3 points, and the total score ranges from 0 to 21 points. The higher the score, the worse the sleep quality (22).

### 2.7. Secondary outcomes

According to the “Guiding Principles of Clinical Research on the Treatment of Insomnia with New Traditional Chinese Medicines”, the criteria for measuring the treatment efficacy in insomnia are established as follows: (1) Clinically cured: Sleep returns to normal, or normal night sleep lasts more than 6 h. There is an increase in deep sleep. Patients are full of energy after wake-up. (2) Markedly effective: sleep is clearly improved, sleep time increases by more than 3 h, and sleep depth increases. (3) Effective: the symptoms are alleviated, and the sleep time increases by <3 h compared with the previous period. (4) Invalid: no obvious improvement or aggravation after treatment. Total effective rate = [(number of clinically cured cases+number of markedly effective cases+number of effective cases)/total number of cases] × 100%. The acupuncturist completed the assessment at the end of treatment (week 2). We assessed the patient's emotional and psychological changes by the total score of SAS and SDS. If a total score of ≥50 indicates anxiety or depression, the higher the score, the more severe the symptoms (23). The acupuncturist completed the assessment before treatment, at the end of treatment (week 2) and during follow-up (weeks 6, 18, and 42).

**Figure 4:**
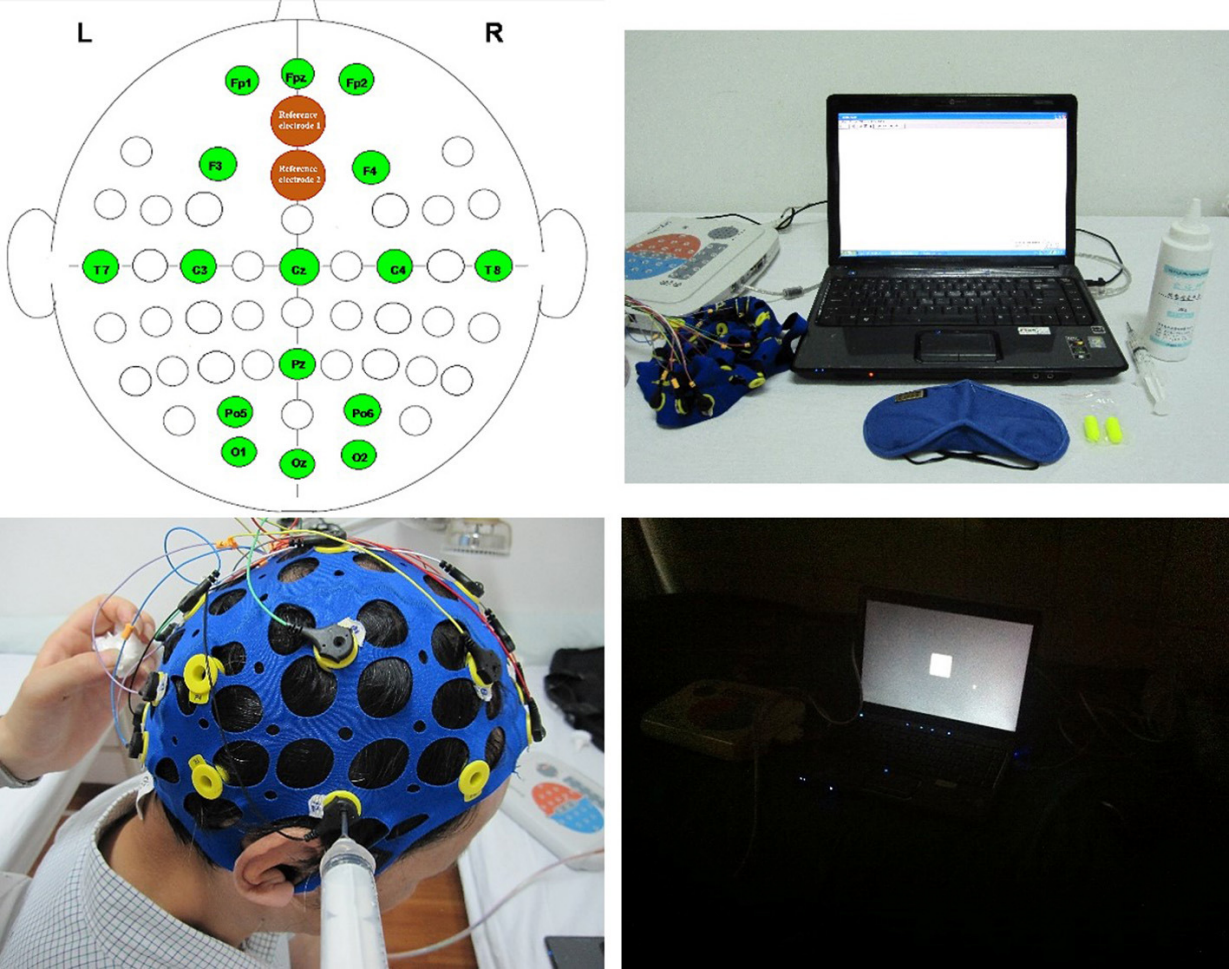
Equipment and data acquisition of SSVEP.

The data collected from SSVEP were used to obtain the potential value through Fourier coefficients (24) (Figure 4). Equipment: HP Compaq Presario V3212 computer, UEA-16FZ EEG amplifier (Beijing Zhongke Xintuo Instrument Co., Ltd.), 16-channel Lycra electrode cap, EEG conductive paste (Jining High-tech Zone Jinnot Medical Gel Factory, batch number: 2013040), dedicated 5 ml conductive paste syringe, 3 M anti-noise earplugs. The steps followed by the acupuncturist for SSVEP data collection are as follows: (i) The acupuncturist asked the patient to sit down and let the patients wear earplugs. Measures were taken to avoid obvious yhead movement of the patients and inability to test questions. The door and windows were closed, and the lights were turned off. (ii) The acupuncturist disinfected the patients' skin of the head, and electrode pads were installed. (iii) Placement region for the brain on the patient's head were in accordance with international standards using electric collection point (2 reference electrodes, 16 recording electrodes). (iv) The acupuncturist injected the electrode paste and then started the scalp resistance test. (v) The acupuncturist put the flash stimulator ~30 cm in front of the patient's eyes and instructed the patient to look at the screen with both eyes. The acupuncturist then started to stimulate vision for 30 s with a frequency between 1 and 16 Hz. After a rest period of 2 s, the next frequency stimulation was started. The measurement was finished after 16 consecutive times. The low potential presents a blue image, which represents EEG inhibition; the high potential presents a red image, which represents EEG excitement. Potential difference = after treatment − before treatment. The acupuncturist completed the assessment before treatment and at the end of treatment (week 2).

### 2.8. Statistical analysis

The baseline characteristics and clinical results of this study were based on intention to treat (ITT) analysis. The included patients received at least 1 treatment and 1 primary outcome measurement. The measurement data was tested for normality first, the unpaired *t*-test or one-way ANOVA test was then used for normal distribution and homogeneity of variance, and the Wilcoxon Signed-Rank or Mann–Whitney U test was used for nonnormality testing. Enumeration data will be tested using χ^2^ analysis. Categorical variables are shown as numbers and percentages. Using the last observation carried forwards method, the missing data of participants who had dropped out were replaced. Using Statistical analysis SPSS^®^ version 22.0, the difference was considered statistically significant at *p* < 0.05.

### 2.9. Quality control

This study was under the supervision of Chengdu University of Traditional Chinese Medicine. Our SSVEP data were analyzed and completed by experts from both the University of Electronic Science and Technology of the University of Life Science and Technology. In addition, a qualified expert in clinical trial research supervised this study.

## 3. Results

### 3.1. Baseline characteristics

A total of 162 patients were approached in this study, and 72 participants met the exclusion criteria. A total of 90 patients met the inclusion criteria (44 in the AA group and 46 in the SA group). We found that the average age of patients with insomnia was 38.77 years and that 64.4% of the subjects were women. The total PSQI scores of the AA group and the SA group in this study were 13.45 ± 1.76 and 13.17 ± 1.91, respectively. There were no significant differences in the SAS and SDS scores between the other two groups (*p* > 0.05) (Table 2).

**Table 2:** Baseline characteristics.

**Variables**	**Active acupuncture (*n =* 44)**	**Sham acupuncture (*n =* 46)**	**χ^2^ or *t*^a^**	***P* ^a^**
Age, mean, y	38.09 ± 13.33	39.41 ± 13.93	−0.460	0.647
Female	28(63.64%)	30(65.22%)	0.03	0.876
Total score of PSQI	13.45 ± 1.76	13.17 ± 1.91	0.724	0.471
SAS score	59.66 ± 6.59	61.24 ± 6.99	−1.102	0.273
SDS score	60.00 ± 4.53	61.59 ± 6.12	−1.393	0.167

Data are presented as the mean ± SD or number (%).

PSQI, Pittsburgh Sleep Quality Index; SAS, Zung Self-Rating Anxiety Scale; SDS, Zung Self-Rating Deprssion Scale.

a Comparison between active acupuncture and sham acupuncture by χ^2^ or unpaired *t*-test.

### 3.2. Primary outcomes

**Figure 5:**
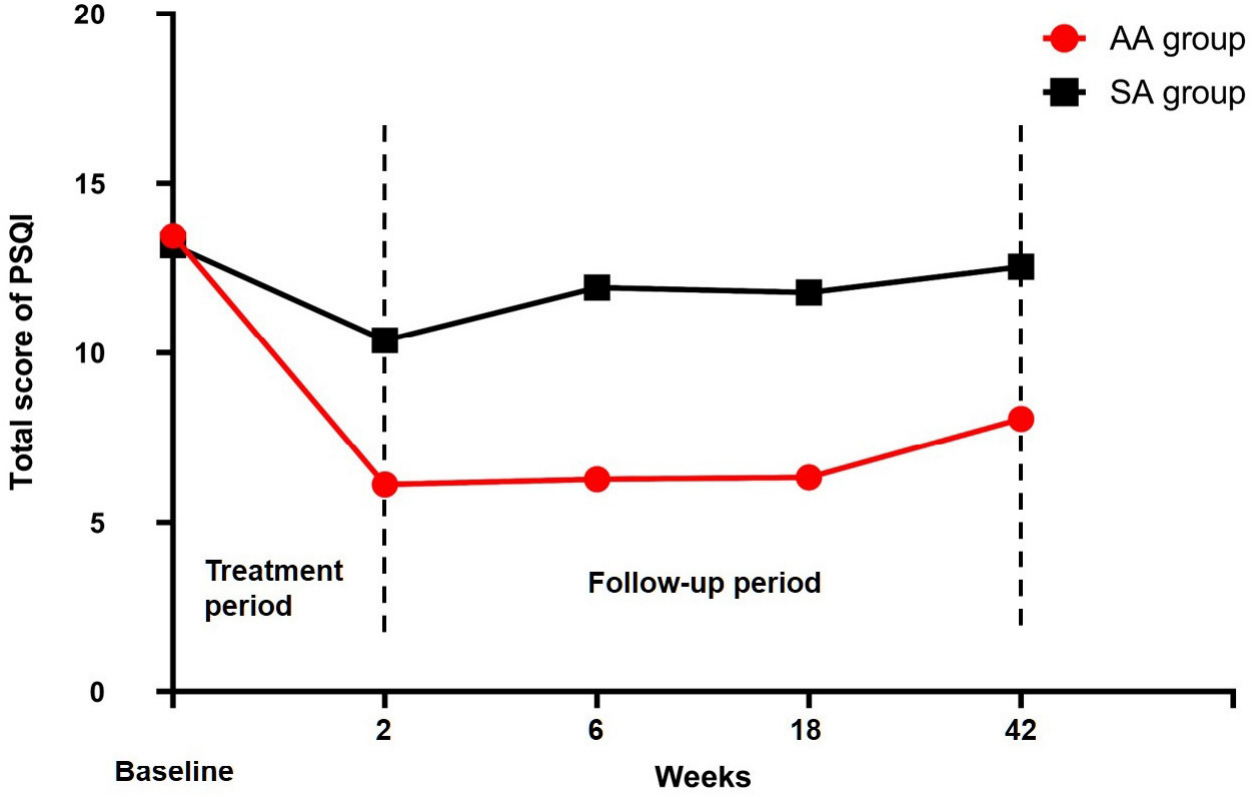
Total PSQI scores were compared between the two groups.

After 2 weeks of intervention, the total PSQI score was 6.11 ± 2.33 in the AA group and 10.37 ± 4.73 in the SA group (*t* = −5.370, *p* < 0.05). During the whole follow-up period, although the total PSQI score of the AA group showed repeated increases to varying degrees, the long-term efficacy of the AA group was still better than that of the SA group (2 weeks: 6.27 ± 1.39 vs. 11.93 ± 3.07, *t* = –11.203, *p* < 0.05/18 weeks: 6.32 ± 2.84 vs. 11.78 ± 2.95, *t* = –8.940, *p* < 0.05/42 weeks: 8.05 ± 3.14 vs. 12.54 ± 2.81, *t* = –7.168, *p* < 0.05) (Table 3, Figure 5). We statistically analyzed 44 patients in the AA group according to the ages of 18–40, 41–55, and 56–65 years. It was found that the clinical effect of acupuncture was improved at any age (all *p* > 0.05) (Table 4).

**Table 3:** Primary outcomes.

**Variables**	**Active acupuncture (*n =* 44)**	**Sham acupuncture (*n =* 46)**	***t* ^a^**	***P* ^a^**
**Total score of PSQI**				
Week 2	6.11 ± 2.33	10.37 ± 4.73	−5.370	0.000^⋆^
Week 6	6.27 ± 1.39	11.93 ± 3.07	−11.203	0.000^⋆^
Week 18	6.32 ± 2.84	11.78 ± 2.95	−8.940	0.000^⋆^
Week 42	8.05 ± 3.14	12.54 ± 2.81	−7.168	0.000^⋆^

Data are presented as the mean ± SD or number (%).

PSQI, Pittsburgh Sleep Quality Index.

a Comparison between active acupuncture and sham acupuncture by unpaired t-test.

⋆ There was statistical significance between the two groups.

**Table 4:** Difference of curative effect in different age segments of active acupuncture group.

**Variables**	**Age, y**			***F* ^a^**	***P* ^a^**

	**18-40 (*****n** =* **25)**	**41-55 (*****n** =* **13)**	**56-65 (*****n** =* **6)**		
**Total score of PSQI**					
Week 0	13.24 ± 1.88	13.92 ± 1.66	13.33 ± 1.51	0.651	0.527
Week 2	5.92 ± 2.22	6.31 ± 2.56	6.50 ± 2.66	0.205	0.815
Week 6	6.20 ± 1.32	6.38 ± 1.45	6.33 ± 1.75	0.079	0.924
Week 18	6.00 ± 2.47	6.92 ± 3.86	6.33 ± 1.75	0.439	0.648
Week 42	7.40 ± 2.75	8.15 ± 2.34	10.50 ± 5.09	2.539	0.091

Data are presented as the mean ± SD or number (%).

PSQI, Pittsburgh Sleep Quality Index.

a Comparison between different age segments by unpaired one-way ANOVA.

### 3.3. Secondary outcomes

**Figure 6:**
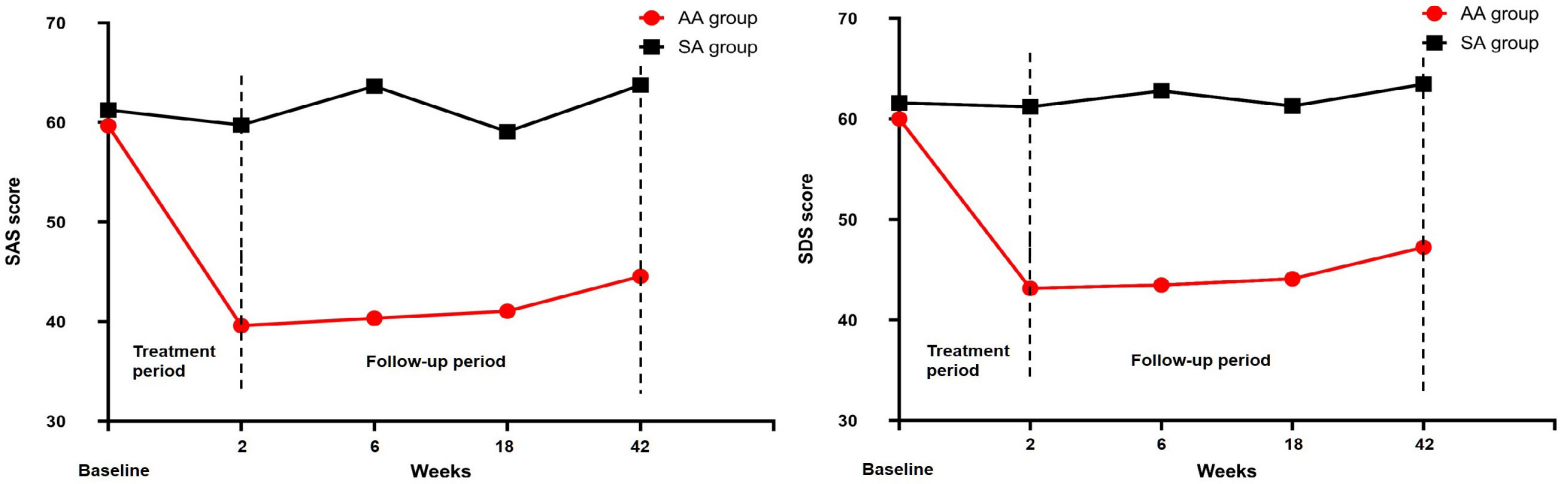
SAS and SDS scores were compared between the two groups.

**Figure 7:**
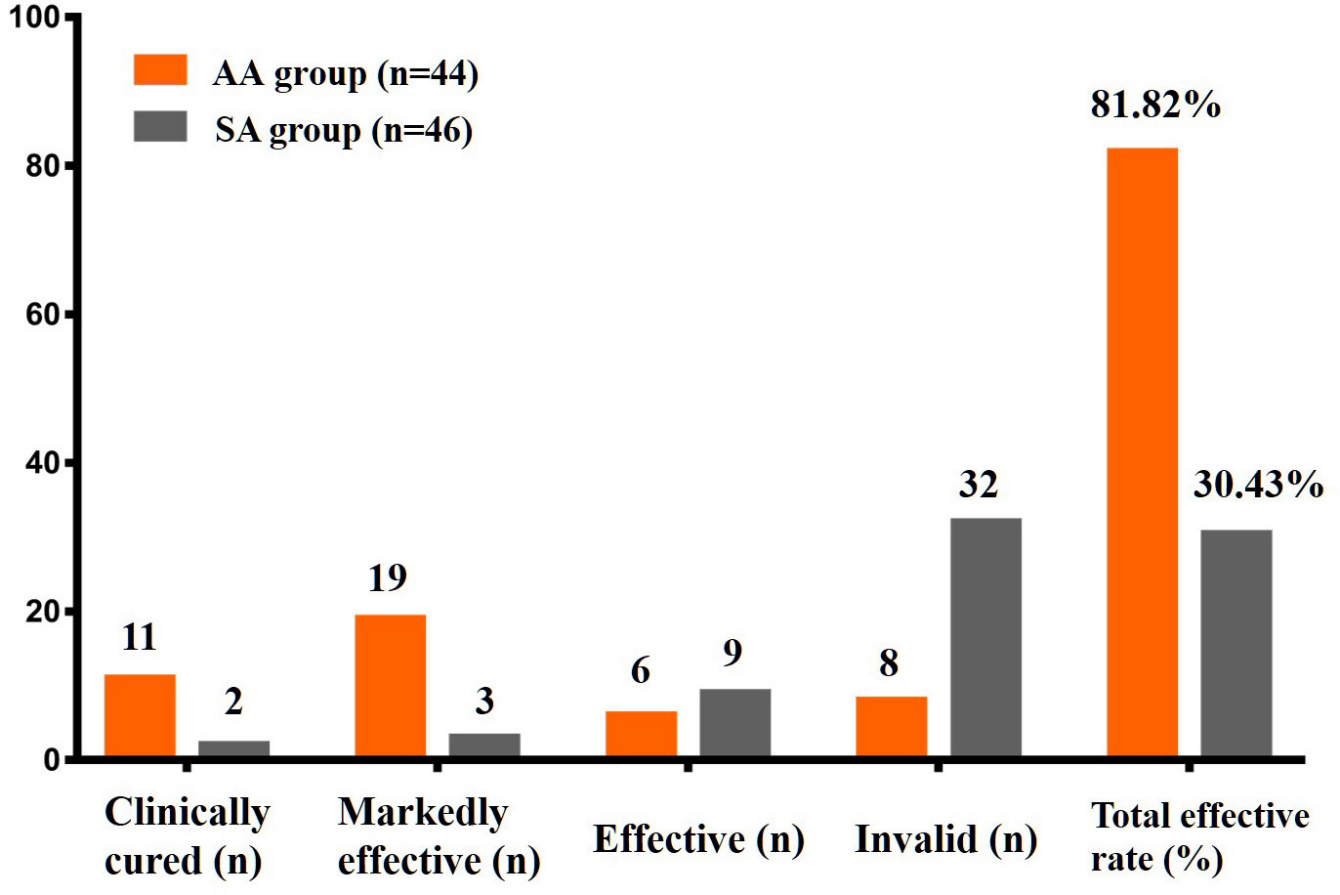
Efficacy level and total effective rate were compared between the two groups.

The SAS score in the AA group decreased to 39.59 ± 5.94 after 2 weeks of intervention, and it was 59.74 ± 6.15 in the SA group (*t* = –15.797, *p* < 0.05). The SDS scores of the two groups were 43.14 ± 10.79 and 61.20 ± 4.40, respectively (*t* = –10.480, *p* < 0.05). The SAS and SDS scores of the two groups were significantly different throughout the follow-up period (Table 5, Figure 6). After 10 treatments, the total effective rate of the AA group was 81.82%, while that of the SA group was 30.43% (χ^2^ = −5.454, *p* < 0.05). The most obvious difference was that the ratio of patients who were clinically cured or markedly improved in the AA group was significantly higher than that in the SA group (11:2; 19:3) (*p* < 0.05). The remaining results are shown in Table 5, Figure 7.

**Table 5:** Secondary outcomes (1).

**Variables**	**AA group (*n =* 44)**	**SA group (*n =* 46)**	**χ^2^ or *t^b^***	***P* ^b^**
**SAS score**				
Week 2	39.59 ± 5.94	59.74 ± 6.15	−15.797	0.000^⋆^
Week 6	40.34 ± 5.18	63.63 ± 6.78	−18.256	0.000^⋆^
Week 18	41.05 ± 5.57	59.07 ± 5.94	−14.828	0.000^⋆^
Week 42	44.55 ± 8.09	63.74 ± 6.67	−12.304	0.000^⋆^
**SDS score**				
Week 2	43.14 ± 10.79	61.20 ± 4.40	−10.480	0.000^⋆^
Week 6	43.48 ± 7.28	62.78 ± 5.17	−14.557	0.000^⋆^
Week 18	44.09 ± 7.62	61.28 ± 4.49	−13.107	0.000^⋆^
Week 42	47.23 ± 10.70	63.46 ± 4.67	−9.392	0.000^⋆^
**Efficacy level**				
Clinically cured	11 (25%)	2 (4.35%)		
Markedly effective	19 (43.19%)	3 (6.52%)		
Effective	6 (13.64%)	9 (19.57%)		
Invalid	8 (18.19%)	32 (69.57%)		
Total effective rate^a^	81.82%	30.43%	−5.454	0.000^⋆^

Data are presented as the mean ± SD or number (%).

SAS, Zung Self-Rating Anxiety Scale; SDS, Zung Self-Rating Depression Scale; AA group, Active Acupuncture Group; SA group, Sham Acupuncture Group.

a Total effective rate (%) = [(number of patients clinically cured + markedly effective + effective)/number of patients] × 100%.

b Comparison between active acupuncture and sham acupuncture by χ^2^ or unpaired *t*-test.

⋆ There was statistical significance between the two groups.

**Figure 8:**
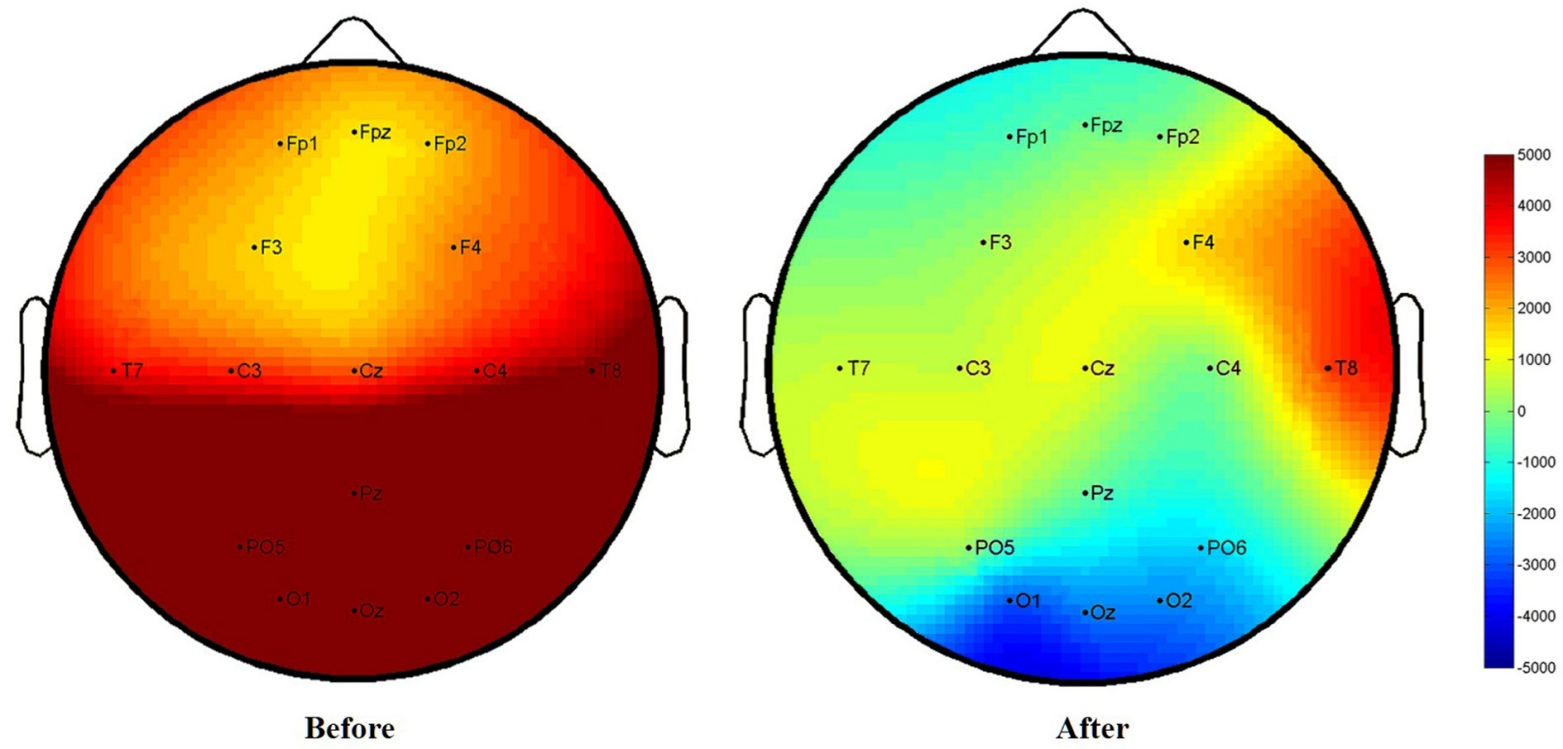
Before and after treatment of the active acupuncture group (SSVEP).

**Figure 9:**
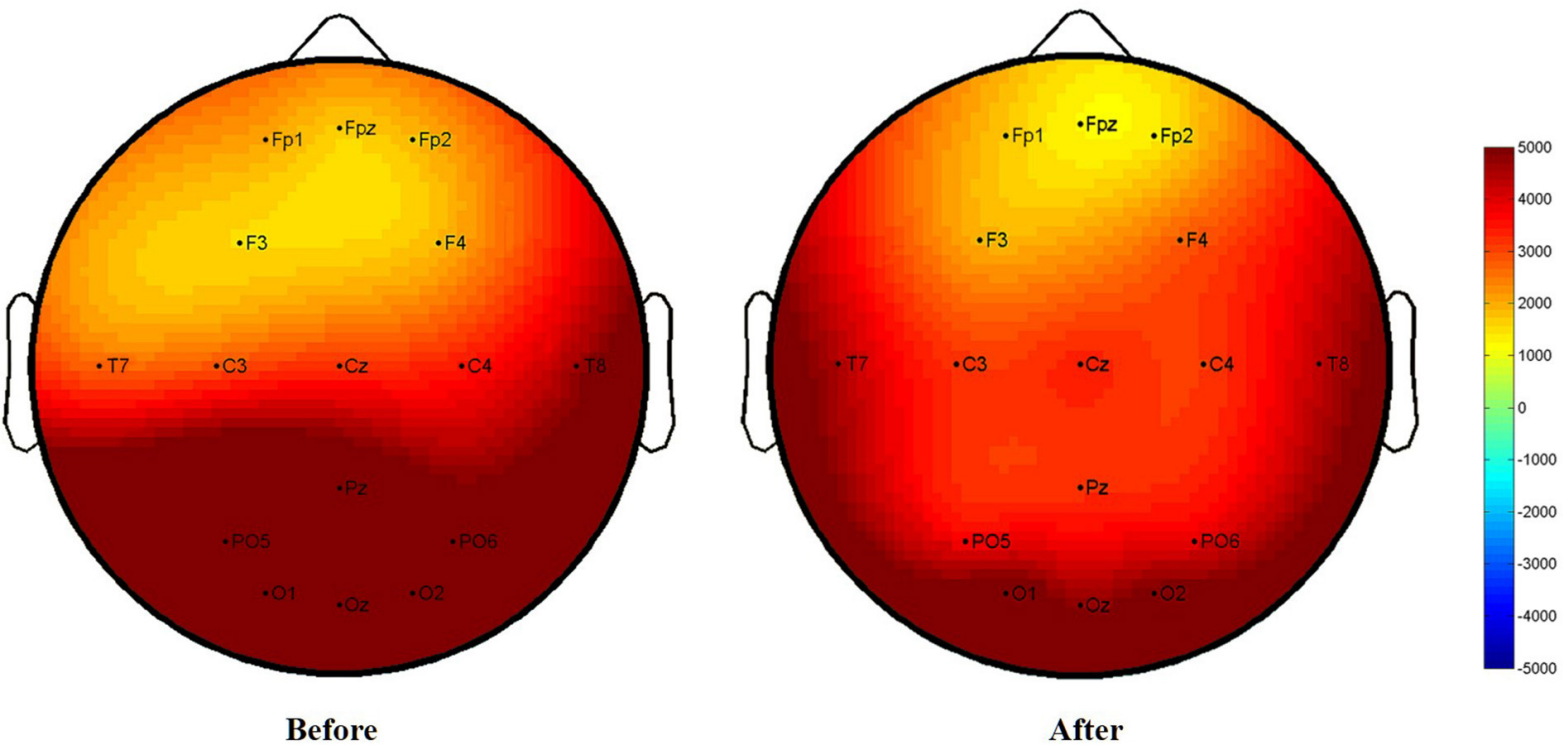
Before and after treatment of the sham acupuncture group (SSVEP).

After the treatment, the brain electrical potential changes in the frontal lobe, right temporal lobe, and occipital lobe in the patients from the AA group were significantly different (*P* < 0.05). Although there was a downwards trend in brain potentials in the SA group, there was no significant difference (*P* > 0.05). When the two groups were compared, there was no difference in the parietal lobe (F4), left temporal lobe (C3), or right temporal lobe (T8) between the AA group and the SA group (*P* > 0.05); however, there were statistically significant differences in brain electrical potential changes in other brain regions (*P* < 0.05). The difference in brain electrical potential changes in specific SSVEP is shown in Table 6, Figures 8, 9.

**Table 6:** Secondary outcomes (2).

**Outcome measure**		**Active acupuncture (*n =* 12)**				**Sham acupuncture (*n =* 12)**					
		**Baseline, 0 week**	**End of treatment,** **2 week**	***z* ^b^**	***P* ^b^**	**Baseline, 0 week**	**End of treatment,** **2 week**	***z* ^b^**	***P* ^b^**	***z* ^c^**	***P* ^c^**
**SSVEP values**											
Brain regions	**Collection point** ^a^										
Frontal lobe	Fp1	1837.48 ± 527.78	−1005.37 ± 534.92	−3.059	0.002^⋆^	1777.95 ± 570.19	1480.37 ± 513.78	−1.274	0.213	−4.161	0.000^⋆^
	Fpz	1678.89 ± 943.97	−1188.33 ± 503.67	−3.059	0.002^⋆^	1364.02 ± 1053.03	1341.60 ± 1023.07	−0.079	0.937	−4.159	0.000^⋆^
	Fp2	1804.94 ± 660.26	−1028.08 ± 614.92	−3.059	0.002^⋆^	1777.18 ± 820.98	1240.12 ± 598.48	−1.571	0.116	−4.164	0.000^⋆^
Parietal lobe	F3	1591.05 ± 863.06	93.01 ± 910.75	−2.824	0.005^⋆^	1164.15 ± 957.03	1553.78 ± 1307.15	−0.393	0.695	−3.004	0.003^⋆^
	Cz	2763.39 ± 2554.71	1106.17 ± 504.41	−2.275	0.023^⋆^	2076.01 ± 579.12	1756.16 ± 762.25	−1.256	0.209	−2.112	0.035^⋆^
	F4	1773.88 ± 764.79	2523.65 ± 303.62	−2.353	0.019^⋆^	1577.77 ± 860.06	1746.99 ± 1159.90	−0.236	0.814	−1.451	0.147
	Po5	6303.71 ± 2553.20	231.15 ± 198.63	−3.061	0.002^⋆^	3988.84 ± 3251.68	3228.09 ± 2223.94	−0.392	0.695	−3.239	0.001^⋆^
	Po6	5347.00 ± 3408.52	−1555.04 ± 229.01	−3.061	0.002^⋆^	5266.82 ± 3862.88	3315.32 ± 1610.80	−1.412	0.158	−4.160	0.000^⋆^
	Pz	5704.06 ± 2708.39	−387.94 ± 215.27	−3.062	0.002^⋆^	3447.92 ± 3475.78	2874.76 ± 1941.17	−0.314	0.754	−4.161	0.000^⋆^
Left temporal lobe	C3	2559.53 ± 1941.23	1659.54 ± 603.56	−1.412	0.158	2590.58 ± 874.17	2249.87 ± 712.02	−1.178	0.239	−1.911	0.056
	T7	2845.63 ± 1529.37	1835.62 ± 380.84	−1.963	0.050	2911.82 ± 1666.74	3840.75 ± 1816.45	−1.256	0.209	−2.693	0.007^⋆^
Right temporal lobe	C4	3309.66 ± 2050.11	−623.20 ± 1410.03	−3.059	0.002^⋆^	2917.48 ± 1048.98	2634.10 ± 1185.69	−0.628	0.530	−4.100	0.000^⋆^
	T8	4118.42 ± 1839.36	3140.23 ± 1351.55	−1.542	0.123	3406.89 ± 1228.21	3847.49 ± 1240.96	−0.785	0.433	−0.925	0.355
Occipital lobe	O1	7741.35 ± 1005.20	−3401.61 ± 1046.62	−3.059	0.002^⋆^	7255.62 ± 1777.63	6658.14 ± 2010.85	−0.549	0.583	−4.159	0.000^⋆^
	Oz	8261.32 ± 5089.34	−2743.79 ± 1801.79	−3.059	0.002^⋆^	8031.33 ± 3639.41	5694.58 ± 3843.58	−1.334	0.182	−4.160	0.000^⋆^
	O2	7989.59 ± 5127.49	−2723.28 ± 1026.82	−3.059	0.002^⋆^	7847.87 ± 3334.90	5689.32 ± 5254.53	−1.099	0.272	−4.159	0.000^⋆^

SSVEP, steady-state visual evoked potentials.

a The collection points of SSVEP are symbols, with no special significance.

b Comparison within-group between active acupuncture and sham acupuncture by Wilcoxon Signed-Rank test.

c Comparison between groups treated with active acupuncture and sham acupuncture by the Mann–Whitney U test.

⋆ The difference between the two groups or within-group was statistically significant.

**Figure 10:**
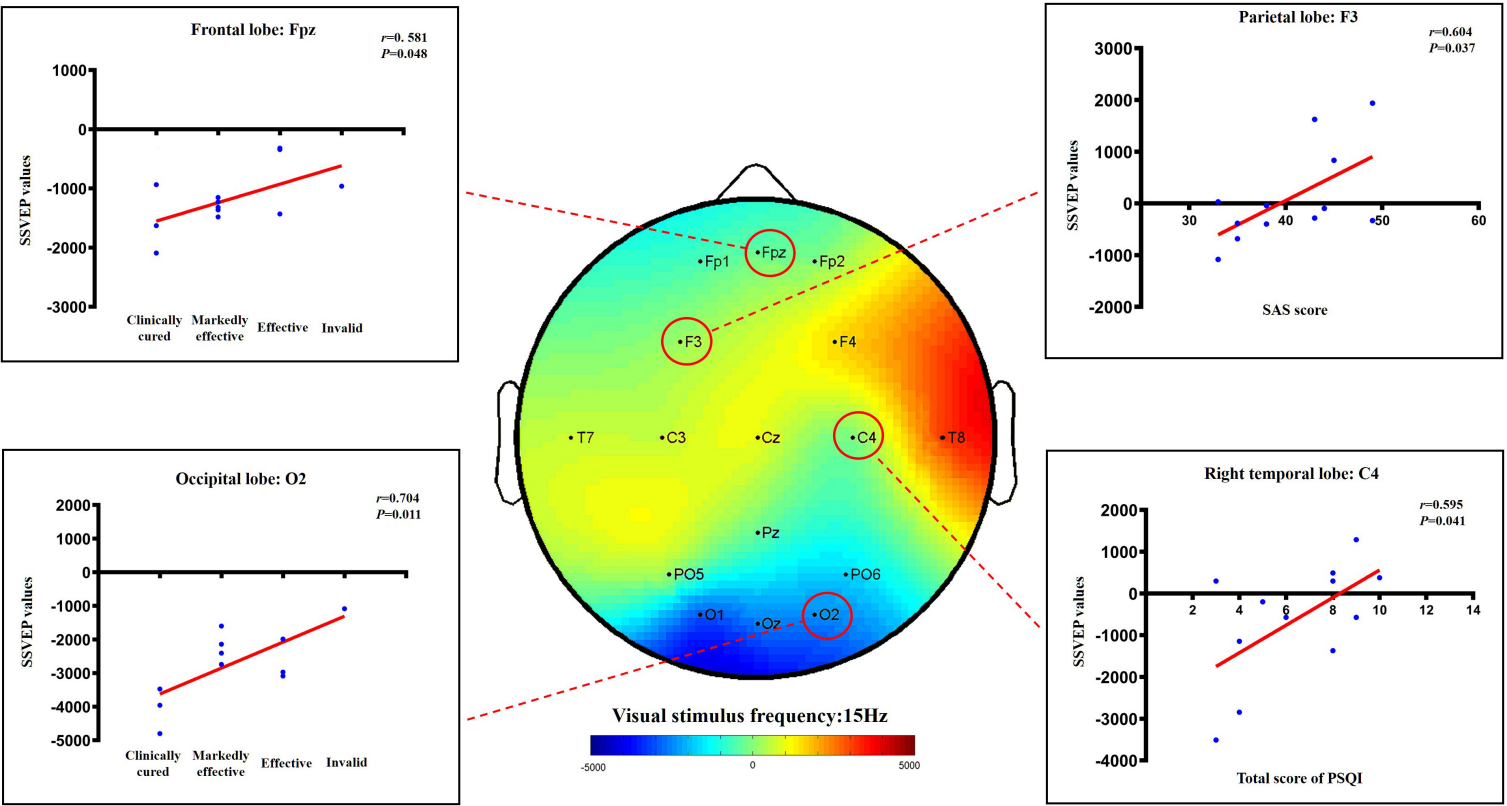
The correlation between sleep improvement and the change in SSVEP values (active acupuncture group).

In addition, our results also demonstrated a certain positive correlation between the total PSQI score, SAS score, efficacy level, and SSVEP value in the AA group. This positive correlation is reflected in the following results: C4 and the total score of PSQI (*r* = 0.595, *P* = 0.041), F3 and SAS score (*r* = 0.604, *P* = 0.037), FPz and the efficiency level of the frontal lobe (*r* = 0.581, *P* = 0.048), and O_2_ and efficiency level of the occipital lobe (*r* = 0.704, *P* = 0.011) (Figure 10).

## 4. Discussion

In our study, we found that, compared with sham acupuncture, active acupuncture can significantly improve sleep quality and the clinical cure rate of insomnia. This effect can be maintained for a long time without being affected by age. At the same time, acupuncture has a significant downregulation effect on the SSVEP of abnormal brain excitement. This downregulation was positively correlated with clinical efficacy. Our study is one of the few that evaluated the observation of acupuncture for insomnia with mood disorders by clinical scales and SSVEP.

Acupuncture has long been used for the treatment of primary insomnia in China. Under current circumstances where oral medication is unfavorable, patients with insomnia often choose acupuncture as an alternative therapy. Compared with the use of drugs for the treatment of insomnia, acupuncture treatment can contribute to significant improvement in the symptoms of insomnia (25). Immediate and sustained effects are the two main manifestations of the curative effect of acupuncture. The onset of the effect of acupuncture on insomnia is a process of transitioning from “quantitative change” to “qualitative change”. The total treatment cycle and the time interval between acupuncture and the onset of symptoms are the two most significant factors influencing the sustained effect. According to the report, to maintain the continuity and stability of the acupuncture effect, it is necessary to compound the number of acupuncture treatments to achieve the best effect (26). Hachul and colleagues reported that there was a 3 point decline in the PSQI score after 10 sessions of acupuncture treatment (27). Fu et al. divided 10 acupuncture treatments into 2 weeks to improve the compliance of patients. After 10 treatments, the PSQI score of the acupuncture group decreased by 8.03 points from baseline, and the PSQI score of the sham acupuncture group decreased by 1.29 points from baseline (28). After 2 weeks of acupuncture treatment, the curative effect will reach a more obvious level and reach the extreme value at 4 weeks. Therefore, a total of 10 treatments were performed in 2 weeks in this study. The advantage is that such accumulation can not only result in therapeutic effects but also avoid the potential effects of excessive acupuncture and increase the body's tolerance.

Acupoints are not only a special place where Qi and blood are infused in the meridian but also the reaction points of the disease and the stimulation points of acupuncture. According to Chinese medicine theory, insomnia results from the vicious cycle of fatigue during the day or from a mental condition of extreme excitement at night. Therefore, this study selected Shenmen, Neiguan, Anmian, and other acupoints that are commonly used for treating insomnia (29). The Anmian is the traditional acupoint used to treat insomnia. Shenmen can calm the heart and spirit. Zusanli affects the spleen and regulates the stomach. Taichong regulates the liver and gallbladder. The Neiguan enlarges the chest and adjusts Qi (30). These acupoints can be used together to calm and soothe the mind and achieve hypnotic induction. The combination of Hegu and Taichong can regulate the liver and Qi. Zhaohai and Shenmai are special meridians and collaterals in the treatment of insomnia and have the advantage of regulating internal organs (31). In traditional Chinese medicine, “deqi” is believed to be the basis for effective results (32). Through acupuncture stimulation of certain acupoints that elicit “deqi” sensations, acupuncture can help restore the normal sleep-wake cycle by correcting the imbalance of Yin and Yang toward harmony.

Our study found that acupuncture treatment does not affect the natural sleep-wake cycle. After 10 acupuncture treatments, the patient's sleep quality and emotional state were significantly improved. In our study, the total PSQI score decreased from 13.45 ± 1.76 to 6.11 ± 2.33. This is still due to the real stimulation of the acupoints, so that the functions of the acupoints can be brought into play. Fu Cong et al. confirmed that the PSQI score decreased by 8.03 points after 10 acupuncture treatments (28). This is consistent with our research results. However, in the SA group, the blunt needle could not penetrate the skin, so the acupoints could not receive useful stimulation. Therefore, the sleep quality of the SA group was still poor. Other test results show that acupuncture can increase the content of serotonin and aminobutyric acid and reduce the level of glutamate (33). Therefore, acupuncture treatment can improve central inhibitory function and thus help people fall asleep (34). It has been reported that the correlation between insomnia and anxiety is 62.2%, and the correlation between insomnia and depression is 59.7%. This shows that insomnia and emotional disorders have a high degree of correlation (35). The frontal lobe is the thinking control center of the brain, which is closely related to the changes in cognitive functions of patients with depression, such as task execution, memory retrieval, information integration, and emotional regulation. Studies have shown that the frontal lobe, temporal lobe and the nerve circuits between them are significantly abnormal in patients with depression (36). It has been observed by resting-state functional magnetic resonance imaging (fMRI) that acupuncture at the Taichong point can inhibit the bilateral frontal lobe and most of the temporal lobe in patients with depression. The change in the frontal lobe may be one of the mechanisms of acupuncture treatment of depression (37). In our study, the SAS and SDS scores of the AA group decreased significantly. Regardless of treatment or follow-up, the improvement in emotional state in the AA group was better than that in the SA group. This situation, we believe, is inseparable from the relief of sleep disorders. Excitingly, the mood disturbances of insomnia patients improved synchronously and were consistent with the quality of sleep. This result has also been confirmed by animal experiments. Cheng Cisong et al. found that electroacupuncture at the Shenmen and Sanyinjiao points can inhibit the overexcitation of the sympathetic adrenal medullary system in rats with insomnia, thereby improving insomnia and alleviating anxiety and other emotional disorders (38). Previous studies have also confirmed that acupuncture can improve the sleep efficiency of patients with insomnia, extend their sleep, and improve insomnia severity (21).

Our results demonstrated that the AA group had better improvement in SSVEP brain potential than the SA group, especially in the frontal and occipital lobes. The frontal lobe is in front of the anterior central sulcus, which accounts for 40% of the cerebral cortex. It is the high-level executive center for brain activity where mental activity occurs. It is inseparably correlated with the symptoms of insomnia and anxiety. The occipital cortex is involved in visual processing. Studies have found that increased gray matter volumes in this cortex are associated with the levels of anxiety severity (39), which may be regulated through the frontoparietal connected networks (40). As the AA group effectively improved sleep quality, the abnormal excitation potential of the cerebral cortex was reduced; the response of the optic nerve in the occipital area was reduced when visual stimulation was received; and the abnormal brain potential of the frontal lobe was also downregulated. Interestingly, this study also found a certain positive correlation between the curative effect, SAS of the AA group and the SSVEP value. When the brain potentials of the frontal, parietal, and occipital lobes were downregulated, sleep quality and mood disorders were effectively adjusted. Using resting-state fMRI, studies have observed that acupuncture may improve sleep quality by regulating the local consistency of the prefrontal cortex and the parietal precuneus (41), which is consistent with our results. Other modern studies have also found that acupuncture can activate the thalamus, frontal lobe and other sleep central functions and can smooth emotions, relieve stress and improve sleep through different neural pathways (42, 43). Because the fibers from the thalamus project to the hypothalamus, the prefrontal lobe of the cerebral cortex, the orbital region or the posterior parietal lobe contact area, this also confirms that acupuncture can play a strong inhibitory role on the SSVEP potential values of the frontal lobe and the occipital lobe brain region, thus effectively improving insomnia and emotional disorders. Acupuncture treatment can not only effectively improve the quality of sleep and emotional state but also improve the quality of life for the subjects.

Visual evoked potentials are widely used in frontier fields such as brain-computer interfaces and are divided into instantaneous visual evoked potentials and steady-state visual evoked potentials. When vision is stimulated by an external stimulus at a constant frequency, the brain produces a response at the same frequency as the external stimulus frequency and its harmonics. The strength of this response can be represented by the voltage signal measured on the scalp, that is, SSVEP. Through cortical homeostasis probe morphology technology, studies have found that anxiety is related to the frontal, temporal, and occipital cortical areas (44). In addition, SSVEP are often used in research on depression, autism and migraine headaches (45, 46). Compared with polysomnography, SSVEP have a strong anti-interference ability and can monitor the dynamic changes in the cognitive process stimulated by external stimuli. Compared with fMRI, SSVEP do not have the problem of haemodynamic delay and are more economical, but they do not have the role of accurately positioning functional brain activation areas (47).

Using SSVEP, we made a new attempt to explore the effects of the brain electrical potential changes following acupuncture for the improvement of sleep quality. However, our study has some limitations, such as a small sample size and a lack of reference to polysomnography. Given the complexity of the disease and the diversification of research approaches, future research can explore the effective combination of SSVEP and fMRI technology to provide a more comprehensive approach to visual analysis of acupuncture treatment for insomnia (48).

In addition, there has been an increase in RCTs of acupuncture for the treatment of insomnia. However, due to variations in methodological quality, previous studies have yielded mixed results. Our study employed the PSD as the main research tool with real needles or blunt needles (49). During operations in which the blunt needle is used, the patient can see the doctor's acupuncture action but does not experience actual needle sensation (50). This is an effective single-blind control method (51, 52). In clinical research studies on acupuncture treatment in low back pain, temporomandibular arthritis, and other systemic diseases, their findings verified the reliability of the Park simulation needle (53, 54). Therefore, to improve the blinding effect, Park analog needles were used in our study in both the acupuncture group and the sham acupuncture group.

## 5. Conclusion

In summary, our research found that acupuncture has a good clinical effect on insomnia patients with emotional disorders and has a significant regulatory effect on abnormally excited brain potentials. Our results provide further evidence for the effectiveness and possible mechanism of acupuncture in treating sleep disorders.

## Data availability statement

The raw data supporting the conclusions of this article will be made available by the authors, without undue reservation.

## Ethics statement

The studies involving human participants were reviewed and approved by the Sichuan Traditional Chinese Medicine (TCM) Regional Ethical Review Committee. The patients/participants provided their written informed consent to participate in this study. Written informed consent was obtained from the individual(s) for the publication of any potentially identifiable images or data included in this article.

## Author contributions

Conceptualization: LZ and YD. Project administration: LZ, RH, and YT. Funding acquisition: YH and NL. Formal analysis: SY and YL. Writing—original draft: LZ. All authors contributed to the article and approved the submitted version.
